# Vaccination Method Affects Immune Response and Bacterial Growth but Not Protection in the *Salmonella* Typhimurium Animal Model of Typhoid

**DOI:** 10.1371/journal.pone.0141356

**Published:** 2015-10-28

**Authors:** Clare L. Kinnear, Richard A. Strugnell

**Affiliations:** 1 Department of Microbiology and Immunology, The University of Melbourne, Melbourne, Victoria, Australia; 2 Department of Biosciences, The University of Melbourne, Melbourne, Victoria, Australia; Leiden University Medical Center, NETHERLANDS

## Abstract

Understanding immune responses elicited by vaccines, together with immune responses required for protection, is fundamental to designing effective vaccines and immunisation programs. This study examines the effects of the route of administration of a live attenuated vaccine on its interactions with, and stimulation of, the murine immune system as well as its ability to increase survival and provide protection from colonisation by a virulent challenge strain. We assess the effect of administration method using the murine model for typhoid, where animals are infected with *S*. Typhimurium. Mice were vaccinated either intravenously or orally with the same live attenuated *S*. Typhimurium strain and data were collected on vaccine strain growth, shedding and stimulation of antibodies and cytokines. Following vaccination, mice were challenged with a virulent strain of *S*. Typhimurium and the protection conferred by the different vaccination routes was measured in terms of challenge suppression and animal survival. The main difference in immune stimulation found in this study was the development of a secretory IgA response in orally-vaccinated mice, which was absent in IV vaccinated mice. While both strains showed similar protection in terms of challenge suppression in systemic organs (spleen and liver) as well as survival, they differed in terms of challenge suppression of virulent pathogens in gut-associated organs. This difference in gut colonisation presents important questions around the ability of vaccines to prevent shedding and transmission. These findings demonstrate that while protection conferred by two vaccines can appear to be the same, the mechanisms controlling the protection can differ and have important implications for infection dynamics within a population.

## Introduction

Typhoid fever, caused by the bacterium *Salmonella enterica* serovar Typhi (*S*. Typhi) causes serious systemic disease in humans with an untreated case fatality rate of 10–20% [1]. The global burden of typhoid has been estimated at 21.7 million infections annually with 217,000 deaths [2]. While strategies for the control of typhoid are reliant primarily on improving sanitation in developing countries, the use of antibiotics and vaccines is becoming more common [3]. Development of resistance to currently available antibiotics has led to a push for increased vaccine usage [4,5]. There are two vaccines currently on the market, first a live attenuated *S*. Typhi strain (Ty21a) that is administered orally, and secondly a Vi polysaccharide subunit vaccine administered parenterally. Both these vaccines are only partially effective with studies finding 16–96% efficacy at 3yrs post vaccination for orally administered vaccines and 55–72% at 1.4–3yrs for injectable vaccines [1,6–14].

Different routes of vaccine administration may result in differing stimulation of the immune system [15,16]. The consequences of any variation in immune response, not only on protection of the individual, but also on how this alters population dynamics such as transmission potential is important to understand in order to develop the most effective vaccine strategies.

The majority of studies on typhoid pathogenesis and immunology rely on mouse-models. *S*. Typhi is human specific and does not cause disease in mice, however recent development of humanised mice models are beginning to allow *S*. Typhi experiments in mice [17–20]. Despite this recent advancement, the majority of studies into typhoid use the closely related *Salmonella enterica* serovar Typhimurium (*S*. Typhimurium), which causes a typhoid-like systemic illness in mice. This system has been used extensively to identify appropriate live-attenuated strains for vaccine use [21–29], and to investigate the immune response required for both primary and secondary protection [30–40]

In order to understand the potential difference in the immune responses elicited by different vaccination methods, it is important to understand the natural route (oral) of infection, and how this differs when immunisation is performed intravenously or by other methods that bypass the gut.

*Salmonella* infection usually occurs through the ingestion of contaminated food or water. The bacteria travel through the gut to the lumen of the small intestine. Here the bacteria cross the epithelial barrier of the gut, either by active uptake by M-cells located predominantly in the Peyer’s patches (PP) [41–45], or through enterocytes [46]. The bacteria then travel through the gut associated lymphoid tissue (GALT) to either the mesenteric lymph nodes (MLN), or directly to the bloodstream by uptake into macrophages, polymorphonuclear phagocytes (PMNs) and dendritic cells (DCs) [32]. Alternatively, bacteria may be engulfed by CX_3_CR1^hi^ mononuclear phagocytes directly from the gut [47,48]. Once inside macrophages, PMNs and DCs, the bacteria travel to the spleen and liver where the bacteria multiply, forming multiple focal lesions which in extreme cases develop in to granulomatous lesions [49]. Transmission of bacteria to new hosts results from large bacterial numbers in the caecum and colon, which are shed in the faeces. The method of colonisation of these organs is yet to be determined, with two alternative, or possibly concurrent, processes being the reseeding of the gut from bacterial populations in the gall bladder or mesenteric lymph nodes, or alternatively from resident populations established in the gut.

Other vaccination methods, which include intramuscular, intravenous and intraperitoneal, bypass all of the initial stages of infection and are delivered directly to the bloodstream [50]. This lack of interaction with the gut and its associated immune cells is likely to result in different stimulation of the immune system. To determine if these differences in the immune response are likely to have an effect on host responses to subsequent infections, an understanding of what components of the immune system are required for control and clearance of salmonella is required.

Low-virulence challenges with *S*. Typhimurium are controlled by the innate immune system (phagocytes and cytokines) [51], and subsequently cleared by CD4^+^ TCR-αβ^+^ T-cells [52]. However, virulent challenges require both early innate responses (macrophages and TNF-α, IL-12, IFN-γ and NK cells) and antibodies and T-cells, which must be primed by immunisation or previous exposure to a sub-lethal infection [53,54].

The role that T-cells and B-cells play in protection against virulent *Salmonella* challenges is still not fully understood, but both components are essential for effective protection. A series of studies using T-cell deficient mice reveal increased bacterial growth, chronic infections and increased host death [53,55]. This is also true of B-cells, where mice lacking B-cells (Igμ^-/-^) show reduced survival to virulent challenge post-vaccination [34]. It is not just T-cells and B-cells in isolation that generate protection, but also the interactions between these components that are important. B-cells are important for effective activation of *Salmonella-*specific T-cells, as CD4^+^ T-cells from B-cell deficient mice have a reduced ability to produce Th-1 type cytokines [36,56]. The reverse is also true, with T-cell-deficient mice, which have impaired T-cell activation or lack proper T-cell/B-cell communication, produce little antibody despite functional B-cells [33,57]. Irrespective of the route taken in generating immunity through vaccination, it is generally believed that the key components required for effective control of secondary virulent salmonella infections are Th-1 type cytokines, specifically TNF-α, IFN-γ, and IL-12 and Salmonella-specific antibodies [31,38,58].

The aim of this study is to determine the effect that different vaccination methods have on immune stimulation and protection from secondary infection in the typhoid mouse model. We used an orally-administered live-attenuated vaccine and an intravenously (IV) administered live-attenuated vaccine. We have characterised the growth and clearance of the vaccine strain after both IV and oral vaccination, as well as shedding of the vaccine strain, systemic and mucosal antibody production, and cytokine serum levels. Further to this, we have determined the level of protection conveyed by each vaccination method, both in terms of growth suppression of the virulent challenge strain, and survival post challenge.

## Materials and Methods

### Ethics Statement

This study was carried out in strict accordance with the approval of The University of Melbourne Animal Ethics Committee (Permit number: 06222). All efforts were made to minimize animal suffering.

### Mice

C57BL/6 mice were bred and housed at the University of Melbourne, Department of Microbiology and Immunology animal facility. All mice were aged 6–8 weeks. When necessary, mice were anesthetised using Penthrane and euthanised by carbon dioxide (CO_2_) asphyxiation.

### Oral vaccination with *S*. Typhimurium

*S*. Typhimurium BRD509, an *aroA* deletion mutant of *S*. Typhimurium SL1344 [59], was used as the vaccine strain in all immunisation experiments. The bacterium was grown, without shaking at 37C for 24hrs in 500ml Luria Bertoni broth (LB) [60] containing 25μg/ml streptomycin. The flask was seeded with 10mls of an overnight culture of BRD509, taken from a glycerol stock. Bacteria from the 500ml culture were pelleted by centrifugation at 13,000 rpm for 15 minutes and resuspended in 800μl phosphate-buffered saline (PBS) to a concentration of approximately 10^11^ cfu/ml. Inoculum counts were confirmed by dilution and plating on LB agar. Mice were administered 200μl (approximately 2 x 10^10^ bacteria) of the inoculum orally, under light anaesthesia, using a 4cm gastric gavage tube. Thirty minutes prior to immunisation, 100μl of 10% v/w sodium bicarbonate in distilled water was administered orally, in a similar manner, to neutralise stomach acidity.

### IV Vaccination with *S*. Typhimurium

Oral vaccine inoculum was diluted to 10^3^ cfu/ml in PBS and 200μl was administered using a 27 gauge needle into the tail vein.

### Oral challenge with virulent *S*. Typhimurium

10ml LB (containing 25ug/ml streptomycin) was subcultured with 50μl of an overnight culture of *S*. Typhimurium SL1344 wildtype (wt) and was grown without shaking at 37C for 24hrs. Bacteria were diluted in PBS to 5 x 10^7^ cfu/ml. Bacterial counts were confirmed by plating on LB agar. Mice were challenged orally with 10^7^ *S*. Typhimurium SL1344 wt in 200μl after pretreatment with sodium bicarbonate.

### Quantification of bacterial colonisation of organs

Mice were sacrificed at the indicated time points or when they were deemed moribund due to significant weight loss and acute physical signs of *Salmonella* infection. Organs (spleen, liver mesenteric lymph node (MLN) and Peyer’s patches (PP)) were collected aseptically and placed in sterile plastic bags. 5ml PBS was added to each sample and samples were homogenised using a Stomacher 80. The number of viable bacteria were quantified by plating serial dilutions of tissue homogenates on LB agar containing streptomycin.

### Bacterial loads–faeces

Mice were placed individually in clean cages with no bedding and 3–5 faecal pellets were collected in a 1.5ml tube. Pellets were weighed and diluted to 100mg/ml in PBS. Pellets were soaked for 1hr on ice then vortexed vigorously for 5mins. The number of viable bacteria per 100mg of faeces were quantified by plating serial dilutions of the faecal homogenate on XLD agar containing 25μg/ml streptomycin.

### ELISA and cytokine sample collection–blood

Blood samples were collected in 1.5ml tubes from mice using tail bleeding. Serum was separated from blood cells by centrifugation at 6,500rpm for 10 minutes, the supernatant was transferred to a clean 1.5ml tube and recentrifuged at 6,500 rpm for 10 minutes. The supernatant was again removed to a clean 1.5ml tube and stored at -20°C until use.

### ELISA sample collection–faeces

Faecal pellets were collected as outlined above and diluted to 100mg/ml in PBS containing 100μg/mL soybean trypsin inhibitor. Pellets were soaked for 1hr on ice, shaken vigorously for 5mins, then centrifuged for 15mins at 13,000rpm. The supernatant was removed to a fresh 1.5ml tube and the protease inhibitor phenyl methyl sulfonyl fluoride (PMSF) was added to a final concentration of 1mM. Samples were stored at -20°C until use.

### ELISA

96-well plates were coated overnight at 4°C with 50μl/well *S*. typhimurium lipopolysaccharide (LPS) (10μg/ml) in PBS. Wells were emptied and blocked for a minimum of 1hr at 37°C with 100μl 1% BSA. Plates were then washed three times with PBS + 0.05% Tween-20 (PBST) and serial dilutions of serum samples in PBST + 0.5% BSA were added to a total volume of 50μl/well. Plates were incubated overnight at 4°C or for 2hrs at 37°C. Plates were washed five times in PBST, then 50μl of HRP-conjugated anti-mouse IgA or IgG diluted 1/1000 in PBST + 0.5% BSA was added to each well and plates were incubated for 2hrs at 37°C or overnight at 4°C. After a final six washes with PBST the plates were developed using 50μl Immunopure o-Phenylenediamine (OPD) with H_2_O_2_ as the substrate. Following 10–15 minutes incubation in the dark, the reaction was stopped using 50μl 2N H_2_SO_4_ and the absorbance was read at 492nm. Endpoint titres were designated as the reciprocal of the dilution of specific antibody that gave an OD_492_ of three times above the negative control reading.

To determine the titre of antibody subclasses in mouse sera, the same procedure as described above was used until the detection step, where 100μl of rat anti-mouse IgG1 or IgG2a specific antibody (diluted 1:1000 in PBST) was added to each well. ELISA plates were incubated with subclassing antibodies for 1 hour at 37C. Unbound antibody was removed by washing plates 10 times in PBST. Bound subclassing reagents were then detected by adding 100μl of goat anti-rat antibody, conjugated to HRP (diluted 1/1000 in PBST), to each well and incubation at 37C for 1 hour. Plates were then washed 10 times with PBST and developed as described above.

### Cytokine CBA Assay

Cytokines, IL-12, IL-10, TNF-α and IFNγ were quantified using the BD Biosciences Cytometric Bead Array (CBA) Mouse Inflammation Kit with modifications as outlined below. Mouse Inflammation Capture Bead suspensions were used at 2μl / bead / test, made up to 50μl with assay diluent. 50μl of the bead suspension was added to 50μl of sample or standard together with 50μl of 1 in 6 dilution of PE Detection Reagents. Samples were incubated for 2 hours at room temperature in the dark then washed with 150μl of wash buffer and centrifuged at 200xg for 5 minutes. The supernatant was discarded and samples were resuspended in 80μl of wash buffer and analysed on a BD FACSCalibur.

### Experimental Design

The effects of different vaccination techniques were examined in a series of three experiments (outlined below) that compared the performance of live *S*. Typhimurium oral and intravenous vaccines in their ability to colonise and elicit immune responses. A further two experiments were performed to asses the ability of these vaccination methods to provide protection from a virulent *S*. Typhimurium challenge.

#### Vaccine colonisation and clearance

Mice were vaccinated either orally (n = 26) or intravenously (n = 27) as described above. At days 7, 14, 28, 42 and 70 five or six mice were sacrificed from each group, and bacterial loads in the spleen, liver, mesenteric lymph nodes and Peyers’ patches were quantified as described above.

#### Faecal shedding and antibody production

Mice were vaccinated either orally (n = 8) or intravenously (n = 7) as described above or left unvaccinated (n = 5). At days 0, 1, 3, 7 and then weekly until day 91, faecal samples were collected as outlined above for both faecal bacterial loads and for faecal IgA quantification by ELISA. On days 1, 7, 14, 21, 28, 42, 56, 70, 84 and 91 post vaccination, blood samples were collected as outlined above for serum IgG quantification by ELISA.

#### Cytokine production

Mice were vaccinated either orally (n = 5) or intravenously (n = 5) as described above or left unvaccinated (n = 5). At days 14 and 28 post vaccination, mice were tail bled as described above and serum samples were prepared for cytokine quantification using a CBA assay as described above.

#### Vaccine protection

Mice were vaccinated either orally (n = 8) or intravenously (n = 9) as described above or left unvaccinated (n = 9). At 10 weeks post vaccination all mice were challenged with a virulent strain of *S*. Typhimurium as outlined above. At day 5 post challenge, mice were sacrificed and bacterial loads in the spleen, liver, mesenteric lymph nodes and Peyer’s patches were quantified as described above.

#### Survival

Mice were vaccinated either orally (n = 14) or intravenously (n = 16) as described above or left unvaccinated (n = 11). At 10 weeks post vaccination all mice were challenged with a virulent strain of *S*. Typhimurium as outlined above. Mice were monitored for signs of severe morbidity (greater than 15% weightloss combined with other acute signs of *Salmonella* infection including hunched posture and oily or ruffled fur) and sacrificed when determined to be moribund. Mice were monitored for 12 weeks post challenge and survival data was recorded for analysis.

### Statistical Analysis

Bacterial counts and antibody reciprocal titres were log10 transformed for normality. Where values were obtained as below the detection limit of the assay a value of half the detection limit was assigned. Bacterial counts from organs were analysed by ANOVA with Tukey HSD post-hoc comparisons performed when necessary. Where normal distributions could not be met due to large numbers of observations below detection limits of assays, non-parametric Van der Waerden tests were performed. IgG antibody responses and cytokine production were analysed using repeated-measures MANOVAs. For cytokine production, levels of cytokines in the serum of unvaccinated mice have been included in figures for comparisons but have not been included in statistical analysis. Statistical analyses were not performed on bacterial counts from faeces and faecal IgA antibody responses as the lack of variation (all values below the detection limit of the assay) in the intravenous vaccine group makes statistical analysis unnecessary and obvious differences can be determined visually. Survival analysis post challenge was performed using the Kaplan-Meier survival platform. Both survival analysis and vaccine protection experiments were performed twice. These data revealed no effect of experiment, and so the data were pooled for all analyses. All analyses were performed in JMP 8.0 (SAS Institute).

## Results

### Vaccine colonisation and clearance

**Fig 1:**
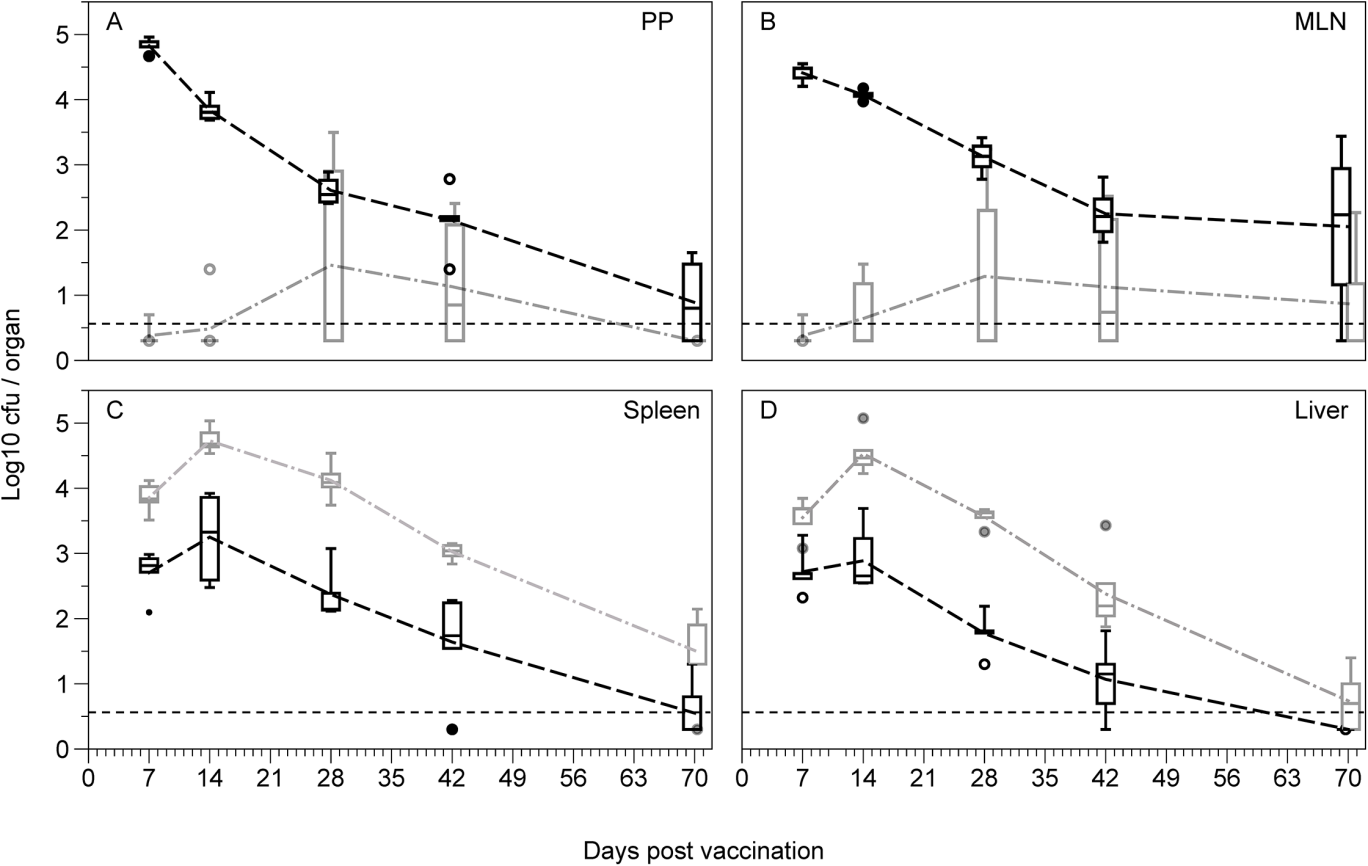
Growth and clearance of the vaccine strain BRD509 following vaccination. Bacterial loads in the **(A)** Peyer’s patches (PP), **(B)** mesenteric lymph nodes (MLN), **(C)** spleen and **(D)** liver of mice over 10 weeks following either oral vaccination with 10^10^ cfu *S*. Typhimurium BRD509 (black), or intravenous vaccination with 10^2^ cfu *S*. Typhimurium BRD509 (grey) (n = 4–7 per group). Dashed grey and black lines represent the means for the IV and oral groups respectively. Boxplots display medians, IQRs and the complete spread of data. Dotted black lines represent the minimum detection limit of the assay (5 cfu). IV vaccination resulted in much higher levels of colonisation in the spleen and liver than oral vaccination, though was rarely detected in the Peyer’s patches or mesenteric lymph nodes.

The organ distribution of the vaccine strain BRD509 was determined after oral or intravenous inoculation. Intravenous and oral administration of the *S*. Typhimurium vaccine strain BRD509 resulted in similar patterns of vaccine growth and clearance over the vaccination period in the spleen, although intravenous administration resulted in 16- to 56-fold higher bacterial load through the peak of vaccine growth (full ANOVA results see Table 1; Fig 1C). The growth and clearance from the liver shows a similar pattern to the spleen, but there is a significant interaction term between vaccination method and day post vaccination, most likely due to the similar bacterial counts between vaccination methods for days 7 as well as the more effective clearance of the IV administered vaccine late (day 28 to 70) (full ANOVA results see Table 1; Fig 1D).

**Table 1:** ANOVA for vaccine colonisation and clearance.

	Spleen		Liver		MLN		PP	
	F-value	P-value	F-value	P-value	F-value	P-value	F-value	P-value
**Full model** ^**a**^	38.234	<0.0001	61.726	<0.0001	20.085	<0.0001	28.655	<0.0001
**Vaccination method** ^**b**^	15.077	0.0004	11.074	0.0018	74.070	<0.0001	112.904	<0.0001
**Day post vaccination** ^**c**^	58.749	<0.0001	102.000	<0.0001	3.286	0.0195	12.397	<0.0001
**Interaction (Vacc x Day)** ^**d**^	1.010	0.4127	4.980	0.0022	8.563	0.0001	16.755	0.0001

^a^ df_between-groups_ = 9, df_within-groups_ = 43

^b^ df_between-groups_ = 1, df_within-groups_ = 43

^c^ df_between-groups_ = 4, df_within-groups_ = 43

^d^ df_between-groups_ = 4, df_within-groups_ = 43

In contrast, analysis of vaccine growth in the gut-associated organs (mesenteric lymph nodes and Peyer’s patches) showed very different patterns of colonisation and clearance based on the method of immunisation. While oral administration of the vaccine strain resulted in colonisation of the mesenteric lymph node (MLN) and Peyer’s patches (PP), intravenous administration rarely resulted in bacteria in these tissues. In the MLN, oral vaccination led to high numbers of bacteria early in the infection, which drops over time, whereas IV vaccination required up to four weeks before bacteria were detected in the majority of mice, and even then only at very low levels (full ANOVA results see Table 1; Fig 1B). This same pattern is seen in the PP (full ANOVA results see Table 1; Fig 1A).

### Faecal shedding

**Fig 2:**
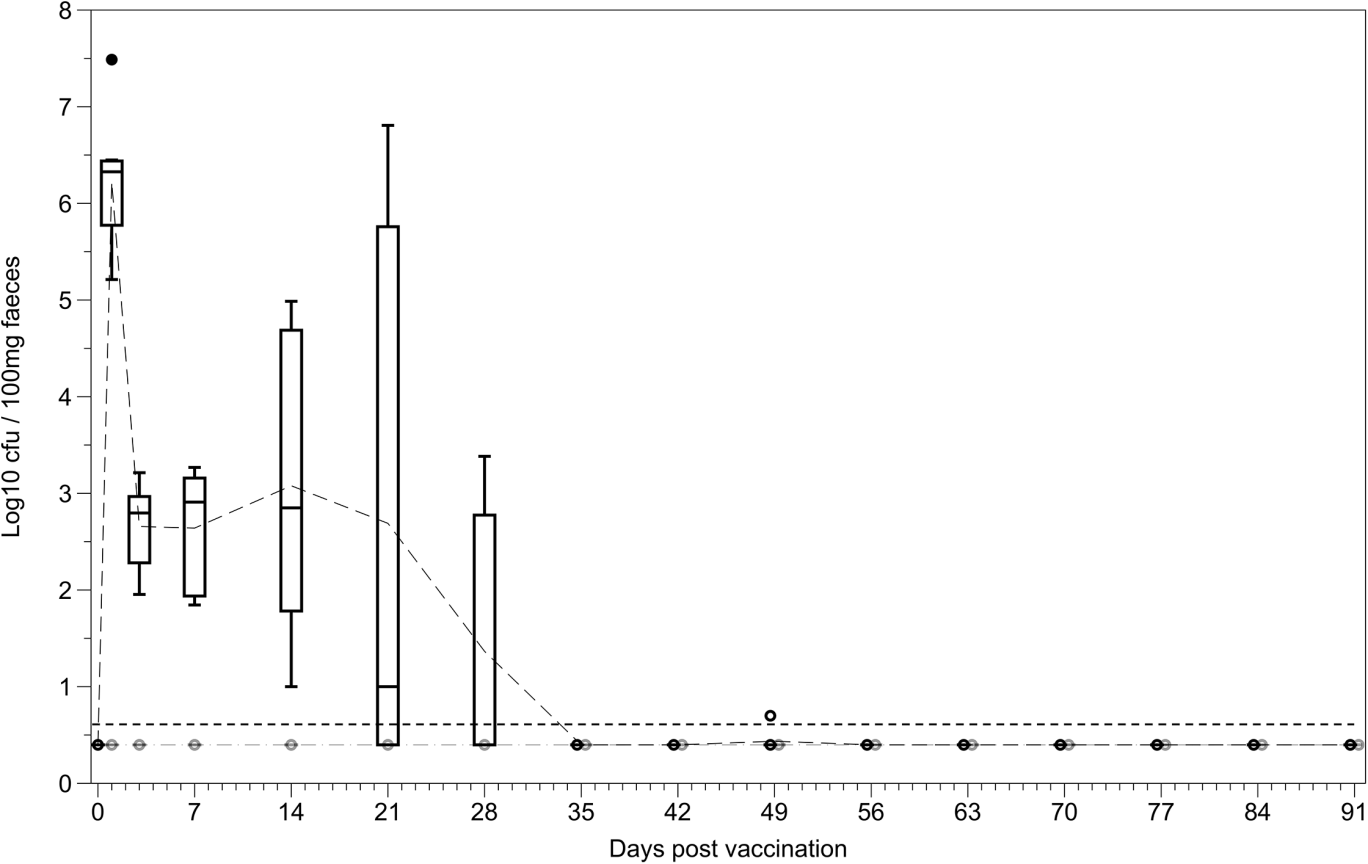
Bacterial shedding of the vaccine strain following oral and IV vaccination with *S*. Typhimurium BRD509. Bacterial loads in the faeces of mice where quantified on days 1, 3 and 7, then weekly for 12 weeks post vaccination with BRD509 either orally (black) or intravenously (grey). Dashed grey and black lines represent the means for the IV and oral groups respectively (n = 5 per group). Boxplots display medians, IQRs and the complete spread of data. Dotted black line represents minimum detection limit of the assay (5 cfu/100mg). Levels of bacteria in the faeces varied greatly between orally vaccinated individuals, but bacteria were generally not detected after 28 days post vaccination. No bacteria were detected in the faeces of IV vaccinated mice at any of the timepoints sampled.

Oral administration of the vaccine strain resulted in faecal shedding of the vaccine strain for up to 5 weeks post vaccination. This was similar to the length of time where significant numbers of bacteria were present in the mesenteric lymph nodes and the Peyer’s patches (Fig 1). Bacterial shedding of the vaccine strain was never observed after IV administration of the vaccine (Fig 2).

### Antibody production

**Fig 3:**
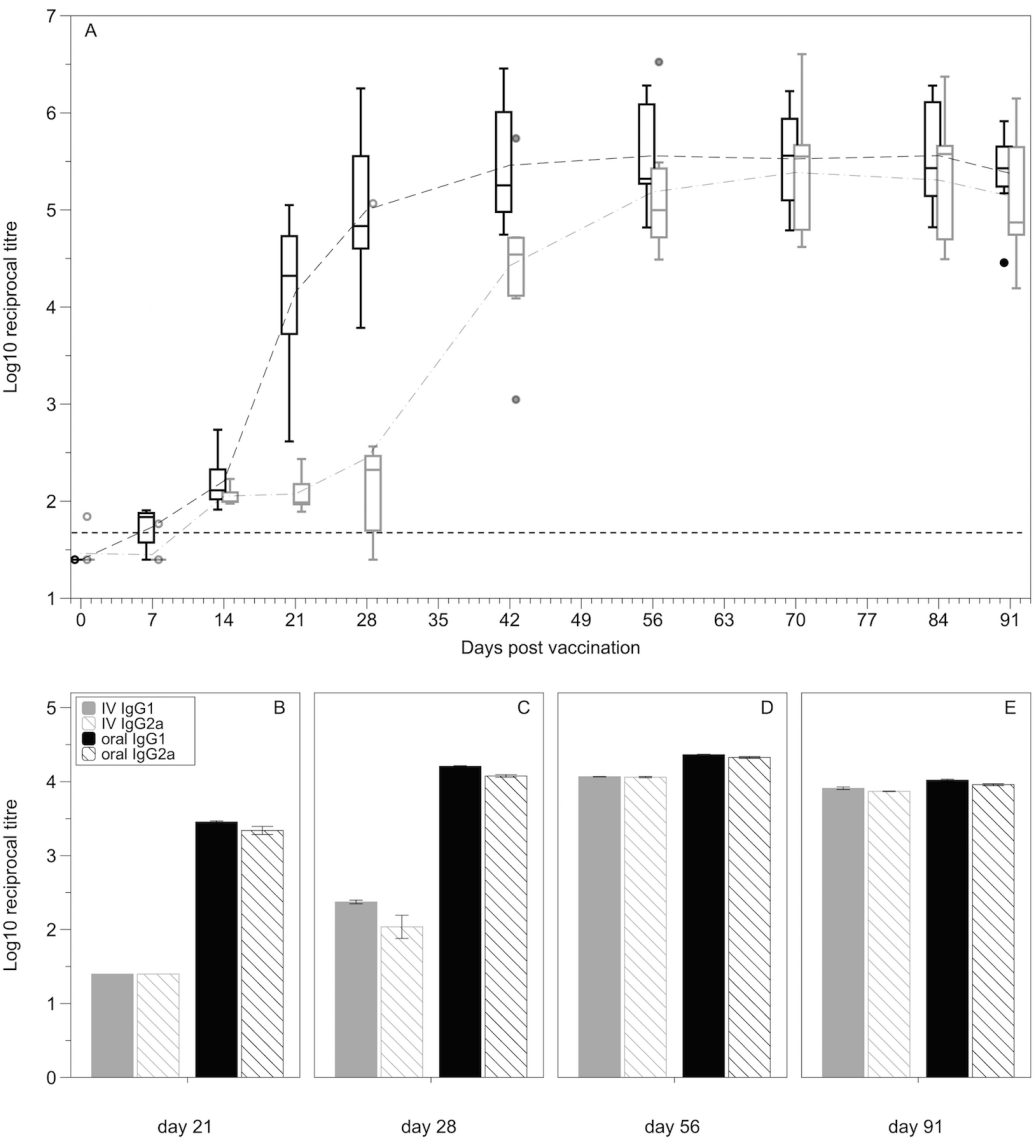
Levels of IgG, IgG1 and IgG2a in serum following IV or oral vaccination. **(A)** anti-LPS IgG antibody levels in the serum of mice vaccinated either orally (black) or intravenously (grey) with BRD509 from day 0 to day 91 post vaccination (n = 6–8 per group). Data are expressed as log_10_ reciprocal titres. Dashed grey and black lines represent the means for the IV and oral groups respectively. Boxplots display medians, IQRs and the complete spread of data. Dotted black line represents the minimum detection limit for the assay (reciprocal titre of 50). **(B-E)** IgG subclassing. Levels of IgG1 and IgG2a in the serum at 21, 28, 56 and 91 days post vaccination either orally or intravenously. Graph represents pooled samples from five mice run in duplicate. Graphs represent mean ± standard error of the mean. There is no obvious quantitative difference between IgG1 and IgG2a levels for either vaccination method at any timepoint.

Both oral and intravenous vaccination resulted in the production of anti-*S*. Typhimurium LPS IgG antibodies. A repeated-measures MANOVA, incorporating both vaccine method and day post vaccination shows a significant effect of both vaccination method and day (Vacc method: F_(1,11)_ = 8.24, p = 0.0152; Day: F_(8,11)_ = 174.90, p<0.0001; Int: F_(8,11)_ = 5.09, p = 0.0666; Fig 3A). The significant vaccination effect is also driven by the delayed antibody production in the intravenous vaccination group compared with the orally vaccinated group. Orally vaccinated mice mounted a *Salmonella*-specific IgG response more rapidly than IV vaccinated mice with significantly higher IgG levels in the serum of orally vaccinated mice at days 21 and 28 post vaccination (post-hoc d21: F_(1,11)_ = 36.6465, p<0.0001; d28: F_(1,11)_ = 13.0880, p = 0.0040). By six weeks post vaccination, both methods elicited a comparable anti-LPS serum IgG antibody response (post-hoc d42: F_(1,11)_ = 1.9895, p = 0.1860), which is maintained over the remainder of the vaccination period. The early serum IgG response seen in the orally vaccinated mice was the result of an increase in both IgG1 and IgG2a (Fig 3B). There is no obvious quantitative difference in IgG1 and IgG2a levels at any of the timepoints (Fig 3B–3E).

**Fig 4:**
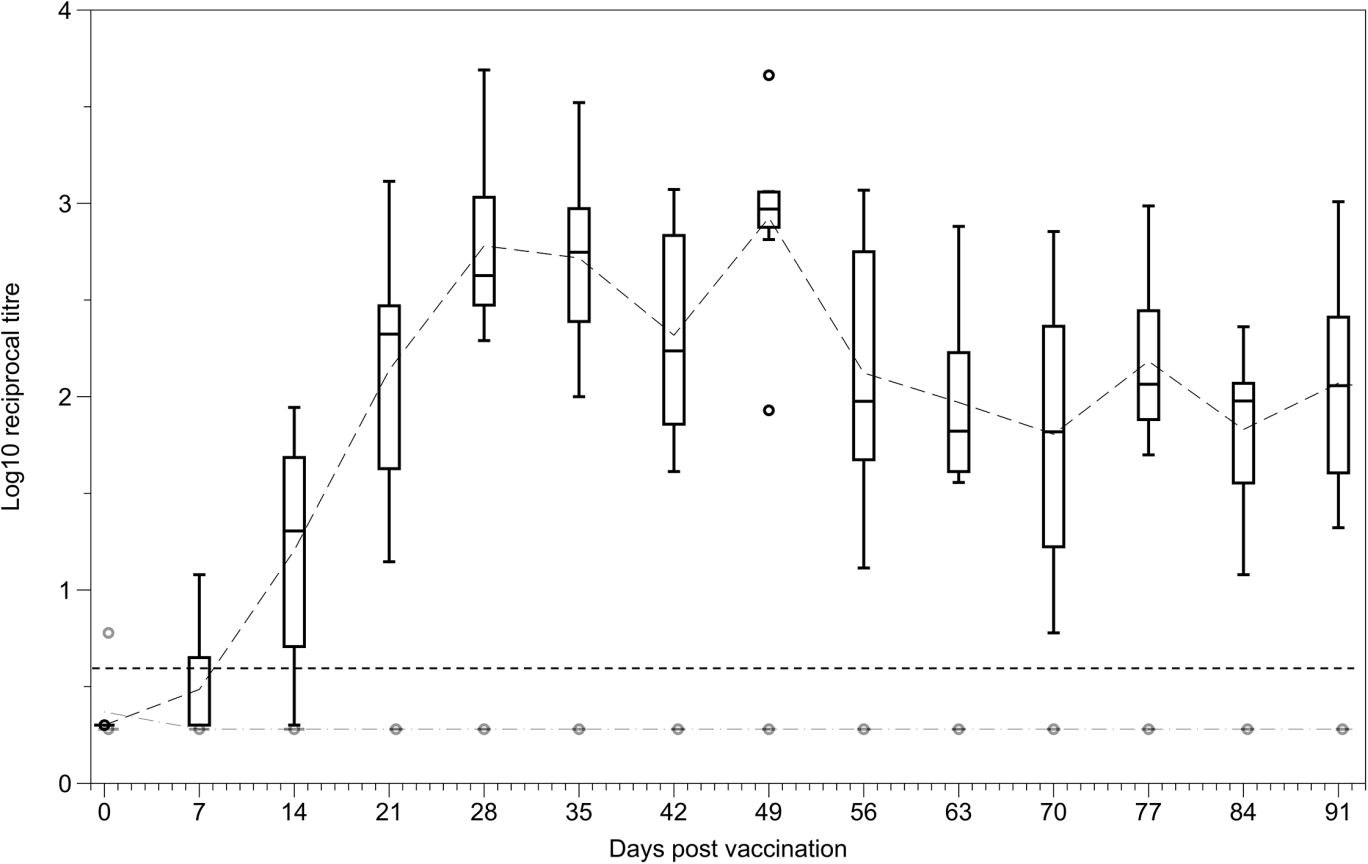
Levels of IgA in the faeces following oral and IV vaccination. Anti-Salmonella LPS IgA antibody levels secreted in the faeces of mice vaccinated either orally (black) or intravenously (grey) from day 0 to day 91 post vaccination (n = 7–8 per group). Data are expressed as log_10_ reciprocal titres. Dashed grey and black lines represent the means for the IV and oral groups respectively. Boxplots display medians, IQRs and the complete spread of data. Dotted line represents the minimum detection limit for the assay (reciprocal titre of 5). Faecal IgA was detected in all orally vaccinated mice by 21 days post vaccination, faecal IgA was never detected in IV vaccinated mice.

Secretory anti-Salmonella LPS IgA antibodies were detected as early as seven days post vaccination in some orally vaccinated mice and were detectable in all orally vaccinated mice by 21 days post vaccination. Faecal IgA was then maintained at a comparable level over the course of the vaccination period. Secretory anti-LPS IgA antibodies were never detected in IV vaccinated mice (Fig 4).

### Cytokine production

**Fig 5:**
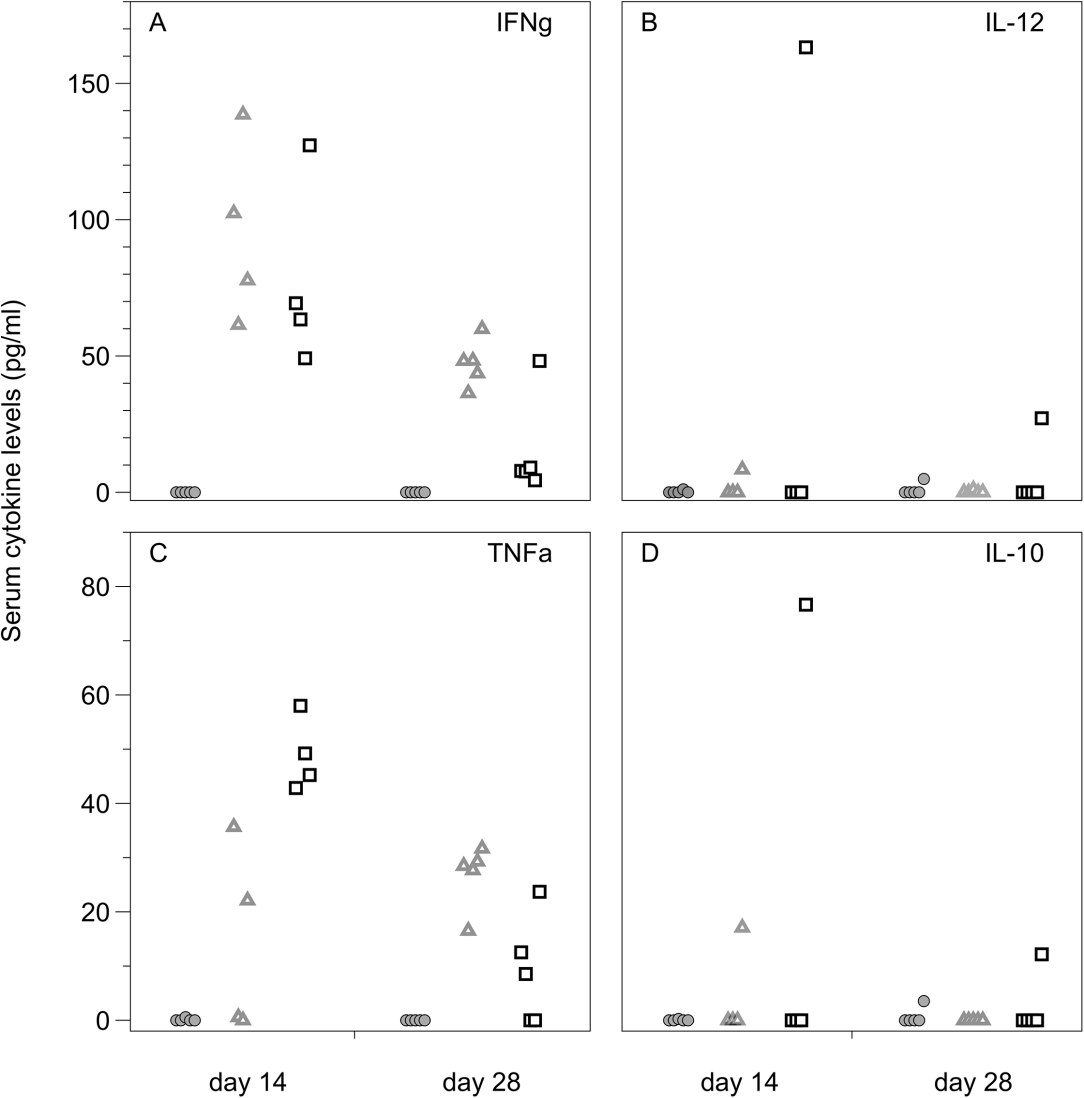
Cytokine levels in the serum of IV vaccinated, orally vaccinated and unvaccinated mice. Cytokine serum levels (pg/ml) at 14 and 28 days post oral (⬜), intravenous (**△**) or no (◯) vaccination. IL-10 and IL-12 were undetectable in almost all mice. An increase in production of IFN-γ and TNF-α was seen in both IV and orally vaccinated mice. For IFN-γ there was no difference between the two vaccination methods. Orally vaccinated mice showed an increase of TNF-α at day 14 which subsequently decreased by day 28. Only some IV vaccinated mice had detectable TNF-α levels at day 14, but TNF-α was detectable in the serum of all IV vaccinated mice at day 28.

IFNγ serum levels did not differ significantly between the two vaccination methods but were significantly reduced at 28 days post-vaccination compared with 14 days post-vaccination (Repeated-measures MANOVA: Vacc method: F_(1,7)_ = 3.07, p = 0.1231; Time: F_(1,7)_ = 24.87, p = 0.0016; Int: F_(1,7)_ = 0.02, p = 0.8807; Fig 5A). IL-10 and IL-12 were undetectable in the serum of the majority of mice at both 14 and 28 days post-vaccination (Fig 5B and 5D).

Analysis of TNF-α levels revealed a significant vaccination method and day post vaccination interaction effect (F_(1,7)_ = 7.59, p = 0.0283), with no main effects (Repeated-measures MANOVA: Vacc method: F_(1,7)_ = 0.36, p = 0.5688; Day: F_(1,7)_ = 1.70, p = 0.2332). Mice vaccinated orally show the highest level of TNF-α at day 14 and then a large reduction in TNF-α by day 28. In mice vaccinated intravenously, TNF-α was only detected in two of five mice at day 14 and in all mice at day 28 but still only at low levels compared to the peak at day 14 for orally vaccinated mice (Fig 5C). From this experiment it is not possible to say if oral vaccination stimulates a greater TNF-α response, as it is possible that a peak in TNF-α levels may just be delayed in IV vaccinated mice and hence not detected in this experiment.

### Vaccine protection

**Fig 6:**
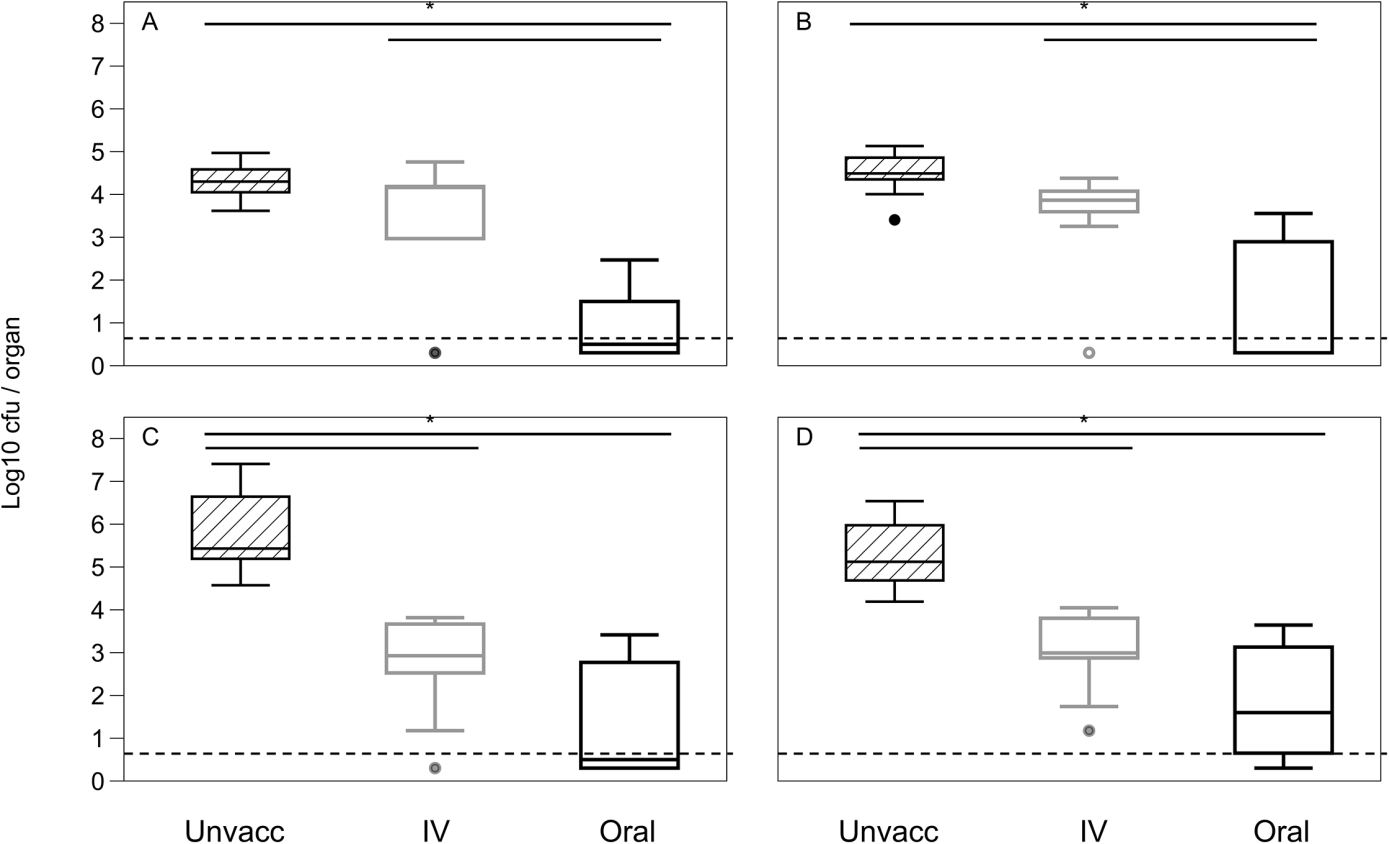
Reduction in colonisation by a virulent pathogen following IV, oral or no vaccination. Bacterial loads in the **(A)** PP, **(B)** MLN, **(C)** spleen and **(D)** liver at day 5 post challenge with a virulent strain following oral (black), intravenous (grey) or no (striped) vaccination (n = 8–9 per group). Dotted line represents the minimum detection limit for the assay (5 cfu / organ). Boxplots display medians, IQRs and the complete spread of data. Data are representative of two experiments. In the spleen and liver both oral and IV vaccination resulted in significant reduction in colonisation by SL1344, with no significant difference between the vaccination methods. In the mesenteric lymph nodes and Peyer’s patches however, oral vaccination resulted in a significant reduction in colonisation by SL1344 but IV vaccination did not. Bars indicate significant difference between groups following unplanned post-hoc t-tests.

Oral and intravenous vaccination led to equal suppression of SL1344 growth in the spleen and liver following challenge with this virulent wild-type *S*. Typhimurium (Spleen: F_(2, 23)_ = 31.41, p < 0.0001; Liver: F_(2, 23)_ = 23.51, p < 0.0001; for post-hoc analysis see Fig 6C & 6D). In the gut associated organs (MLN and PP), there is also an overall vaccination effect (Van der Waerden: MLN: χ^2^ = 16.58, df = 2, p = 0.0003; PP: χ^2^ = 11.75, df = 2, p = 0.0028; Fig 6C and 6D). Oral vaccination resulted in reduced colonisation following a virulent challenge, but unlike in the systemic organs, IV vaccination did not lead to a significant reduction in colonisation of these organs compared to unvaccinated mice (for post-hoc analysis see Fig 6A and 6B).

### Survival

**Fig 7:**
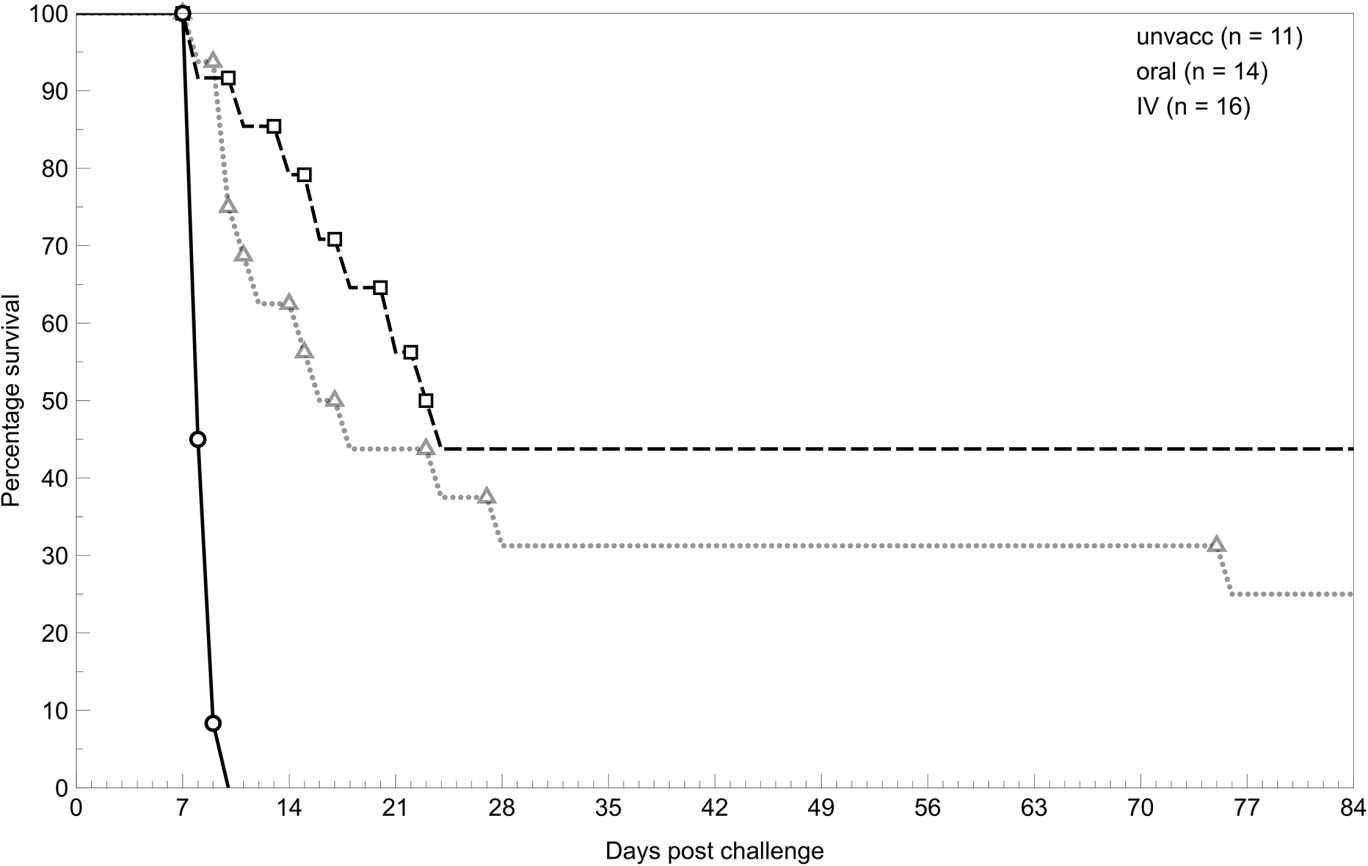
Survival of mice given a virulent challenge following IV, oral or no vaccination. Mice were either orally vaccinated with 10^10^ BRD509 (⬜) or IV vaccinated with 10^2^ BRD509 (**△**) 10 weeks prior to challenge or unvaccinated (◯). Data are representative of two experiments. Both IV and oral vaccination increased the survival of mice compared to the unvaccinated group. There was no significant difference in survival between orally and IV vaccinated mice.

Both methods of vaccination resulted in an increased survival following challenge with virulent *S*. Typhimurium compared with unvaccinated mice. There was no significant difference in the survival times of mice vaccinated either intravenously (25% survival), or orally (44% survival) over a 12-week period, post virulent challenge (IV/oral survival: Wilcoxon: χ^2^ = 1.21, df = 1, p = 0.2709; Log-Rank: χ^2^ = 1.25, df = 1, p = 0.2637; Fig 7).

## Discussion

Comparative studies of immune responses to vaccines predominantly focus on differences in the vaccine strains [27–29,61,62], or the effectiveness of adjuvants or specific antigens [63,64], with only a handful providing any insight into the more simple modifications like method of delivery [65–67]. This study demonstrates that administering the same vaccine via different routes can result in significant differences in immune stimulation. In the murine typhoid model, these differences did not impact survival from secondary infections.

The most obvious difference in immune response detected in this study is in the induction of specific secretory IgA (SIgA) in orally, but not IV vaccinated mice. These results indicate that early gut interactions are important for production of secretory IgA. IV vaccinated mice that have minimal to no bacterial colonisation of the PP and MLN, also fail to generate a detectable secretory IgA response. This is consistent with studies showing that colonisation of the PP is required for the induction of a SIgA response [65].

The differences between vaccination methods in immune stimulation are also reflected in the outcome of a secondary challenge with a virulent strain. Our data shows that oral vaccination resulted in a significant reduction in colonisation in gut-associated organs from a secondary challenge compared with IV vaccinated mice. In IV-vaccinated mice, the colonisation levels in these organs are comparable to unvaccinated mice. This effect might be due to SIgA as previous studies have shown that mice that have no secretory antibody response (pIgR^-/-^) are more susceptible to infections with virulent strains [68], and also that infections with *Salmonella* strains that are unable to be bound by SIgA result in higher levels of colonisation in systemic organs than SIgA sensitive strains [69]. However, in a study by Uren *et al*., immunised pIgR^-/-^ mice show similar protection against secondary colonisation of the gut-associated organs as C57BL/6 mice, indicating that it is not SIgA alone that is responsible for the reduced gut colonisation [70].

Another interesting difference in immune stimulation between the two vaccination methods is the delay in development of a serum IgG response in IV vaccinated mice compared with orally vaccinated mice. Surprisingly, the higher levels of colonisation in the systemic organs in IV vaccinated mice by the vaccine strain did not result in greater induction of specific antibody. Instead, interactions of orally administered bacteria with the immune system in the gut-associated organs and GALT (gut-associate lymphoid tissue), as occurs during oral vaccination, appears to lead to more rapid development of serum antibodies, with peak serum IgG levels reached up to 4 weeks faster (in half the time) by oral vaccination.

Despite the differences discussed in immune stimulation and protection from colonisation of the gut-associated organs, there is no difference in survival of mice challenged with a virulent strain following IV or oral vaccination. This lack of difference in survival is also reflected in the similar levels of protection from colonisation by secondary infection in the systemic organs (spleen and liver). While oral vaccination can prevent colonisation of systemic organs [69], our data show that it is not essential for preventing systemic colonisation and doesn’t increase survival.

We have highlighted the differences in immune response between our vaccination methods, but the lack of difference in protection in systemic organs and overall survival most likely results from the many similarities in immune response between the two groups. Firstly there is no significant difference in stimulation of the cytokines INFγ, IL-10, IL-12 and TNFα, which are deemed essential for control of secondary infections [31,38,58]. Despite the delay in serum IgG stimulation in IV vaccinated mice, again there is no difference in serum IgG titres between the vaccination methods at the time of challenge. These data indicate that while oral vaccination had significant implications for early control and secondary colonisation of the gut-associate organs, both vaccination methods were equally capable of generating an immune response to control systemic infection and protect the vaccinated individual.

While both vaccination methods provide comparable protection for the vaccinated individual in murine typhoid, the difference in the ability of both the vaccination strain and the secondary challenge strains to colonise the gut in IV and orally vaccinated mice has significant implications for the health of others in the population. First, the colonisation of the gut and the resulting shedding of the vaccine strain from orally vaccinated mice increases the potential for transmission of the vaccine strain to other individuals in areas with poor sanitation. This may be either a positive, as it helps to generate immunity in the population as a whole, or a negative, as it puts immunocompromised individuals at serious risk. As the infectious dose required to transmit the vaccine strain is significantly higher than the level of shedding we see here (Fig 2), it seems unlikely that transmission would be a significant issue.

The local response elicited by oral but not IV vaccination prevents bacteria from colonising not only the PP and MLN but also the small intestine and the colon (see S1 Fig). As bacteria travel out of the colon in the faeces, bacterial numbers in the colon are likely a proxy for the number of bacteria being shed and the risk of transmission from that individual. While orally vaccinated mice have a minimal chance of transmitting secondary infections, as they have almost no detectable bacteria in the gut, IV vaccination provides no reduction in gut colonisation from a secondary infection. This means that while IV vaccinated individuals gain reasonable protection against disease, they are just as likely as unvaccinated individuals to transmit a virulent pathogen.

Our data highlight that while two vaccines may be equally capable of controlling an infection and protecting vaccinated individuals from disease, this does not mean that they are of equal merit for use as control mechanisms in populations. Here, our two vaccination methods have the potential to result in significant differences in the spread of pathogens between individuals in a population. The implications of these differences for infection dynamics are unknown and vital for predicting population wide implications of vaccine strategies.

## Supporting Information

S1 Fig Reduction in colonisation by a virulent pathogen (SL1344) following IV, oral or no vaccination in the gut. Bacterial loads in the contents of the a) small intestines and b) colon at 48hrs post challenge with a virulent strain following oral (black), intravenous (grey) or no (striped) vaccination (n = 4 per group). Data are represented as means + standard error of the mean. Dotted line represents the minimum detection limit for the assay (5cfu / organ).
(TIFF)
