# Supplementary figures and images for: Vaccination Method Affects Immune Response and Bacterial Growth but Not Protection in the Salmonella Typhimurium Animal Model of Typhoid
Source: PLoS One. 2015 Oct 28;10(10):e0141356. doi: 10.1371/journal.pone.0141356 (PMC4625024; doi:10.1371/journal.pone.0141356)

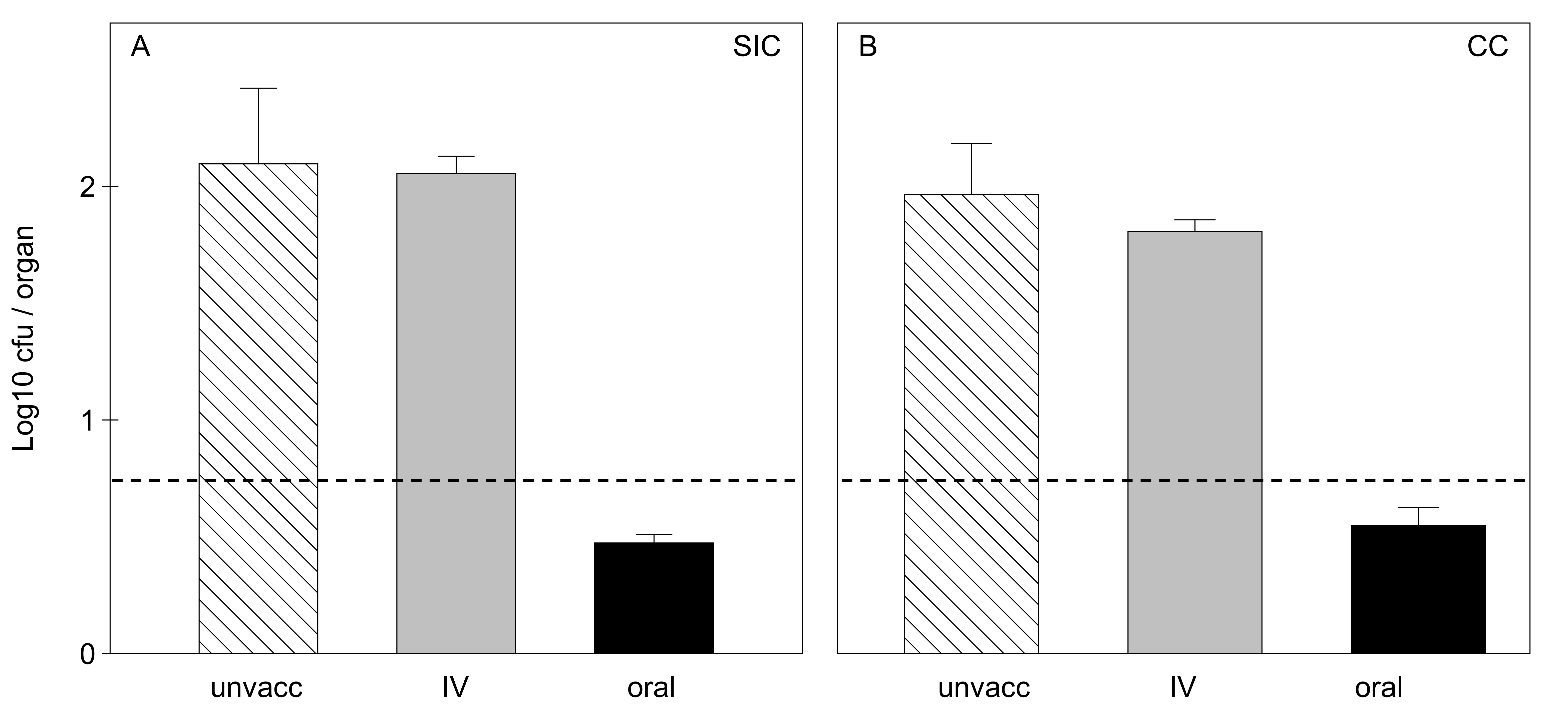

Supplement: S1 Fig — Bacterial loads in the contents of the a) small intestines and b) colon at 48hrs post challenge with a virulent strain following oral (black), intravenous (grey) or no (striped) vaccination (n = 4 per group). Data are represented as means + standard error of the mean. Dotted line represents the minimum detection limit for the assay (5cfu / organ). (TIFF) [file pone.0141356.s001.tiff]
